# Mesoporous Oxidized Mn-Ca Nanoparticles as Potential Antimicrobial Agents for Wound Healing

**DOI:** 10.3390/molecules29132960

**Published:** 2024-06-21

**Authors:** Qianfeng He, Hui Yuan, Youshen Bu, Jiangshan Hu, Olagoke Zacchaeus Olatunde, Lijie Gong, Peiyuan Wang, Ting Hu, Yuhang Li, Canzhong Lu

**Affiliations:** 1 State Key Laboratory of Structural Chemistry, Fujian Institute of Research on the Structure of Matter, Chinese Academy of Sciences, Fuzhou 350002, China; xmheqianfeng@fjirsm.ac.cn (Q.H.); xmyuanhui@fjirsm.ac.cn (H.Y.); buyousen@163.com (Y.B.); hjs5231@163.com (J.H.); olatunde@fjirsm.ac.cn (O.Z.O.); ljgong@fjirsm.ac.cn (L.G.); wangpeiyuan@fjirsm.ac.cn (P.W.); huting148@163.com (T.H.); 2 College of Chemistry, Fuzhou University, Fuzhou 350116, China; 3 Xiamen Key Laboratory of Rare Earth Photoelectric Functional Materials, Xiamen Institute of Rare Earth Materials, Haixi Institutes, Chinese Academy of Sciences, Xiamen 361021, China; 4 Key Laboratory of Functional and Clinical Translational Medicine, Fujian Province University, Xiamen Medical College, Xiamen 361023, China

**Keywords:** mesoporous silicon, reactive oxygen species, antibacterial, wound healing

## Abstract

Managing chronic non-healing wounds presents a significant clinical challenge due to their frequent bacterial infections. Mesoporous silica-based materials possess robust wound-healing capabilities attributed to their renowned antimicrobial properties. The current study details the advancement of mesoporous silicon-loaded MnO and CaO molecules (HMn-Ca) against bacterial infections and chronic non-healing wounds. HMn-Ca was synthesized by reducing manganese chloride and calcium chloride by urotropine solution with mesoporous silicon as the template, thereby transforming the manganese and calcium ions on the framework of mesoporous silicon. The developed HMn-Ca was investigated using scanning electron microscopy (SEM), transmission electron microscope (TEM), ultraviolet-visible (UV-visible), and visible spectrophotometry, followed by the determination of Zeta potential. The production of reactive oxygen species (ROS) was determined by using the 3,3,5,5-tetramethylbenzidine (TMB) oxidation reaction. The wound healing effectiveness of the synthesized HMn-Ca is evaluated in a bacterial-infected mouse model. The loading of MnO and CaO inside mesoporous silicon enhanced the generation of ROS and the capacity of bacterial capture, subsequently decomposing the bacterial membrane, leading to the puncturing of the bacterial membrane, followed by cellular demise. As a result, treatment with HMn-Ca could improve the healing of the bacterial-infected wound, illustrating a straightforward yet potent method for engineering nanozymes tailored for antibacterial therapy.

## 1. Introduction

Dealing with chronic non-healing wounds presents a substantial clinical hurdle that is associated with many causes, including diabetes mellitus (DM), vascular disease, aging, and bacterial infection [1,2,3]. Bacterial infections have become the chief cause of patients’ deaths [4]. Antibiotic agents are used to prevent bacteria from breeding. However, overdose and improper use of antibiotics could lead to an increase in antibiotic resistance, which is a frequent occurrence during extended therapy for addressing chronic wounds (for diabetic foot conditions, venous ulcers, and pressure wounds) [5,6]. In addition, treatment of drug-resistant bacteria typically involves higher doses of antibiotics and a variety of costly medications that may offer reduced efficacy, increased toxicity, and induce unwanted side effects [7,8]. Hence, the development of an alternative therapeutic approach to alleviating infection occurrence and enhancing the process of wound healing has become a major concern worldwide [9,10].

Conventional wound dressings [11] (such as films, bandages, tapes, cotton wools, and gauzes) have limited therapeutic efficacy and numerous side effects, including renal and hepatic toxicity, which restrict their application [12]. Conversely, nanoparticles (NPs) have been considered to be widely used in wound dressings owing to their distinctive physicochemical, optical, and biological properties in recent years [13,14,15]. Reports have shown that metal oxide NPs, including Au, Ag, Si, Fe, MnO_2_, and ZnO NPs, exhibit remarkable attributes such as minimal in vivo toxicity and bacteriostatic/bactericidal effects [16,17,18,19,20,21,22]. Incorporation of CaO nanoparticles into metal oxide NPs will improve their effectiveness and minimize drawbacks by triggering bacterial calcium death and accelerating coagulation, thus contributing to bacteriostasis [23,24,25,26,27]. Also, MnO molecules have Fenton-like catalytic properties within cells [28,29,30]. Mn^2+^ acts as a catalyst to facilitate the production of reactive oxygen species (ROS) to trigger oxidative stress and antimicrobial function [31,32,33]. It has been found that manganese can promote neovascularization and synergistic antibacterial effects with other metal oxide nanoparticles [34,35,36,37]. Nanocomposites, a class of materials with sustainability and biodegradability, are suitable candidates to develop antimicrobial agents [38]. To date, many potent antibacterial nanocomposites have been developed, such as MnFe_2_O_4_ nanocomposites and Fe_3_O_4_@Chitosan-AgNP nanocomposites, which could inhibit the growth of Bacillus cereus and Escherichia coli [39].

Porous and mesoporous materials have been widely used in the loading of antimicrobial materials or drugs [40]. For example, mesoporous silica nanospheres (MSNs) hold a great prospect for their particular physicochemical prospect [41], including a broad inner surface and volume, controllable size, ease of modification, and thermal stability [42]. In addition to these advantages, MSNs have abundant silica hydroxyl groups on the surface, which are the optimal choice to combine with other nanomaterials to endow the materials with antimicrobial abilities [43]. Moreover, unlike traditional antibiotics, which lead to several side effects, MSNs show a broad antimicrobial spectrum and are not easily prone to drug resistance [44]. These nanoparticles are now recognized as a desirable choice of antibiotics and potential alternatives for multi-drug resistance (MDR) microbial contamination [45,46].

Inspired by the above investigations, we prepared MnO and CaO-loaded mesoporous silica nanoparticles (HMn-Ca) in the present study. Due to mesoporous silica’s exceptional adhesive capacity, the nanomaterial demonstrates high affinity for adhering to cell membranes of methicillin-resistant *Staphylococcus aureus* (MRSA) and extended-spectrumβ-lactamases-producing *Escherichia coli* (ESBL-EC) [47]. Furthermore, MnO molecules can catalyze the production of ROS, thus killing the multidrug-resistant bacteria [48,49]. Calcium oxide will further lead to a calcium imbalance, causing the death of the bacteria. Moreover, CaO nanoparticles may reduce the extravasation of blood following wound injury and enhance wound closure [50]. In both Gram-negative and Gram-positive bacteria, excessive calcium and ROS can induce damage to the bacterial structure and organization, resulting in the inhibition of bacterial growth in both types of bacteria [51]. Calcium and ROS are crucially involved in both the propagation and execution stages of necrotic cell death, leading directly or indirectly to damage to proteins, lipids, and DNA. This ultimately leads to the breakdown of cell integrity [52,53]. Here, the HMn-Ca prepared in this study demonstrates the capability to concurrently inhibit the growth of MRSA and ESBL-EC while also improving the healing of the bacterial-infected wound. Essentially, the present research offers a dependable instrument for the treatment of multidrug-resistant bacterial infections and related chronic non-healing wounds.

## 2. Results and Discussion

### 2.1. Synthesis and Characterization of HMn-Ca Nanoparticles

**Figure 1:**
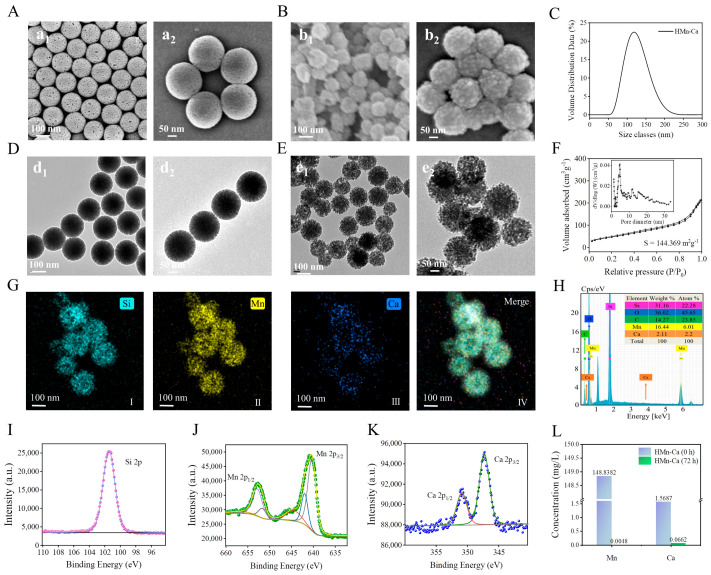
Synthesis and characterizations of HMn-Ca. (**A**) SEM image of MSNs (the scale for a_1_ is 100 nm, while the scale for a_2_ is 50 nm). (**B**) SEM image of HMn-Ca (b_1_ and b_2_ are depicted under different scales). (**C**) Particle size distribution of HMn-Ca. (**D**)TEM image of MSNs (d_1_ and d_2_ are observed under different scales). (**E**) TEM image of HMn-Ca (e_1_ and e_2_ with different scales). (**F**) N_2_ adsorption–desorption isotherms and the corresponding pore size distribution curves (inset) of HMn-Ca. (**G**) Elemental mapping images and (**H**) SEM-EDS of HMn-Ca. (**I**) XPS patterns of HMn-Ca. The blue curve is the theoretical binding energy peak of Si^2+^, and the purple dot is the experimental data, the two data are consistent. (**J**) XPS patterns of Mn. The yellow curve is the theoretical binding energy peak of Mn^2+^, and the green dot is the experimental data, which are basically consistent. (**K**) XPS patterns of Ca^2+^, the theoretical data are green and red curves, and the experimental data are blue dots. (**L**) ICP of fresh and post-HMn-Ca to confirm the stability of HMn-Ca.

The template MSNs were synthesized by the hydrolysis of tetraethyl orthosilicate in the presence of cetyltrimethylammonium bromide (CTAB, as the structure-directing agent) and triethylmethylammonium (Et_3_N, which provides an alkaline catalytic environment). As indicated by SEM, the prepared MSNs displayed high specific surface areas and typical mesoporous structures with uniform pore structures and dimensions (Figure 1A). These characteristics make MSNs suitable for the surface adsorption and nucleation of nanoparticles. Therefore, the MSNs were employed as the template for material synthesis. We further loaded MnO and CaO molecules onto MSNs as described in Section 4.2. The obtained HMn-Ca nanoparticle showed a hollow and floral morphology with an average size of 100 nm, as well as uniform pore channels and dimensions with discernible mesoporous structures (Figure 1A,D). The result in Figure 1C was corroborated by SEM and TEM images, indicating that the particle size distribution of HMn-Ca primarily centers around 122.42 nm. Additionally, the synthesis method of HMn-Ca possesses advantages in terms of its simplicity, ease of operation, and subsequently enabling large-scale productions.

We also characterized the formation of HMn-Ca via TEM. Both MSNs and HMn-Ca exhibited divergent pores and a high specific surface area, but HMn-Ca exhibited a more defined pore size and incorporated partial hollow structures with a uniform distribution of Si, Mn, and Ca in the materials (Figure 1B,E). We hypothesized that this phenomenon is attributed to the weak alkaline nature of urotropine, which acted as an etchant and selectively removed silicon oxide during the synthesis, resulting in a partially hollow structure and an increment in the specific surface area of HMn-Ca. We observed a more pronounced hollow in HMn-Ca through mapping analysis (Figure 1G). This disparity arises from the utilization of different voltages in TEM, specifically 80V for TEM imaging and a higher voltage of 200 kV for mapping (Figure 1G). The increased voltage, owing to the relatively soft structure of silicon oxide, resulted in the fragmentation of silicon oxide cavities during mapping, consequently exaggerating the apparent size of the cavities in the mapping analysis.

Moreover, the BET analysis also verifies the formation of the mesoporous architecture in HMn-Ca. Figure 1F displays the N_2_ adsorption–desorption curves for the HMn-Ca. The pore volume of the material, calculated using the Brunauer–Emmett–Teller (BET) method and derived from the measured N_2_ adsorption–desorption data, reveals the surface area of HMn-Ca is 144.369 m^2^/g with a pore volume of 0.3299 cm^3^/g. In accordance with the IUPAC standard, the obtained isotherm is identified as a type IV isotherm with a hysteresis loop, indicating mesoporous characteristics. The elevated specific surface area of HMn-Ca can be attributed, in part, to the substantial loading of nano-sized particles of MnO and CaO. Additionally, the presence of cavities resulting from the surface etching by urotropine contributes to the increased surface area. Based on this finding, we postulate that our material exhibits superior adsorption capacity. This structural foundation establishes HMn-Ca as a promising antibacterial material.

In addition, the formation of HMn-Ca was also confirmed by EDS analysis and XPS analysis. EDS analysis indicated a successful loading with MnO and CaO molecules at a percentage of 16.44% and 2.11%, respectively (Figure 1H). The composition of HMn-Ca was then verified by XPS. As depicted in Figure 1I, the binding energy maxima of 101.45 eV was assigned to Si 2p. The peaks at 652.7 and 641.3 eV in the Mn 2p spectrum were attributed to Mn 2p_1/2_ and Mn 2p_3/2_, respectively (Figure 1J). Furthermore, at binding energies of 351.4 eV and 347.4 eV, the typical peaks corresponding to Ca 2p_1/2_ and Ca 2p_3/2_ were observed (Figure 1K). These findings indicated the formation of HMn-Ca.

Finally, we tested the stability of HMn-Ca in biological conditions. As indicated in Figure 1L, it was observed that the concentration of Mn was 0.0048 mg/L, while that of Ca was 0.0662 mg/L, representing only 0.01% of the total solution concentration. This result suggested that HMn-Ca is stable in biological conditions; only a small amount of Mn and Ca would be released in the aqueous solution.

### 2.2. HMn-Ca Promotes Antimicrobial Activity In Vitro

**Figure 2:**
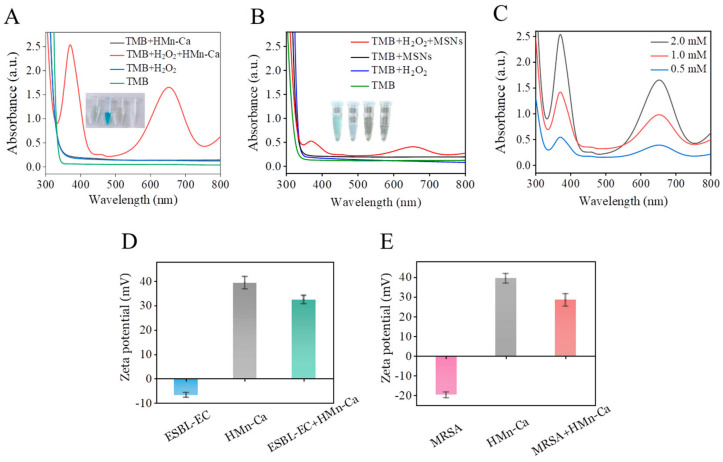
Characterizations of HMn-Ca. Changes in absorbance at 652 nm of POD-like activity are measured by (**A**) HMn-Ca and (**B**) MSNs. (**C**) Absorbance changes at 652 nm of POD-like activity by HMn-Ca are dependent on the dose. Zeta potential of (**D**) ESBL-EC and (**E**) MRSA after exposure to PBS or HMn-Ca.

MnO NPs have Fenton-like catalytic characteristics that can promote the production of ROS and cause oxidative stress in cells [54]. Thus, we further investigated the peroxidase (POD)-like nature of HMn-Ca, which has the capacity to catalyze endogenous hydrogen peroxide and generate ROS, utilizing 3,3′,5,5′-tetramethylbenzidine (TMB) as a ROS capturer. TMB has the capability to trap ROS and produce a bluish substance exhibiting maximum absorbance at 652 nm. As shown in Figure 2A, the remarkable POD-like activity of HMn-Ca is reflected by its high absorbance at 652 nm, and the intensity of this absorbance exhibited a positive correlation with the concentration of H_2_O_2_ (Figure 2C). These results suggested POD-like activity and the potential antibacterial effects of HMn-Ca. Moreover, the template MSNs had no such effects (Figure 2B), indicating the POD-like activity of HMn-Ca derived from metal NPs, probably MnO NPs.

Next, we studied the bacterial capturing capacity of HMn-Ca by zeta potential tests, which were widely used in assessing the interactions between materials and bacteria [55]. By characterizing the variation in bacterial surface potential before and after material binding, such as the increase in negative potential values following the binding of positively charged materials with bacteria [56], we can clarify the interaction between HMn-Ca and bacteria. Antibiotic-resistant Gram-positive MRSA and Gram-negative ESBL-EC were used to test the binding affinity of HMn-Ca to the bacteria. For both bacteria, a highly negative surface charge is obtained at a neutral pH value (Figure 2D,E), due to the abundance of negatively charged glycoproteins present on the cell membrane surface. In contrast, the HMn-Ca nanoparticle has a positive surface charge (38.9 ± 2.4 mV) in the same condition (Figure 2D,E). However, the zeta potentials of E. coli and MRSA solutions were greatly decreased after incubation with HMn-Ca (Figure 2D,E), indicating that HMn-Ca may adhere to the surface of bacteria via electrostatic attraction. Furthermore, the high specific surface area of HMn-Ca may also contribute to the adhesion of the bacteria.

**Figure 3:**
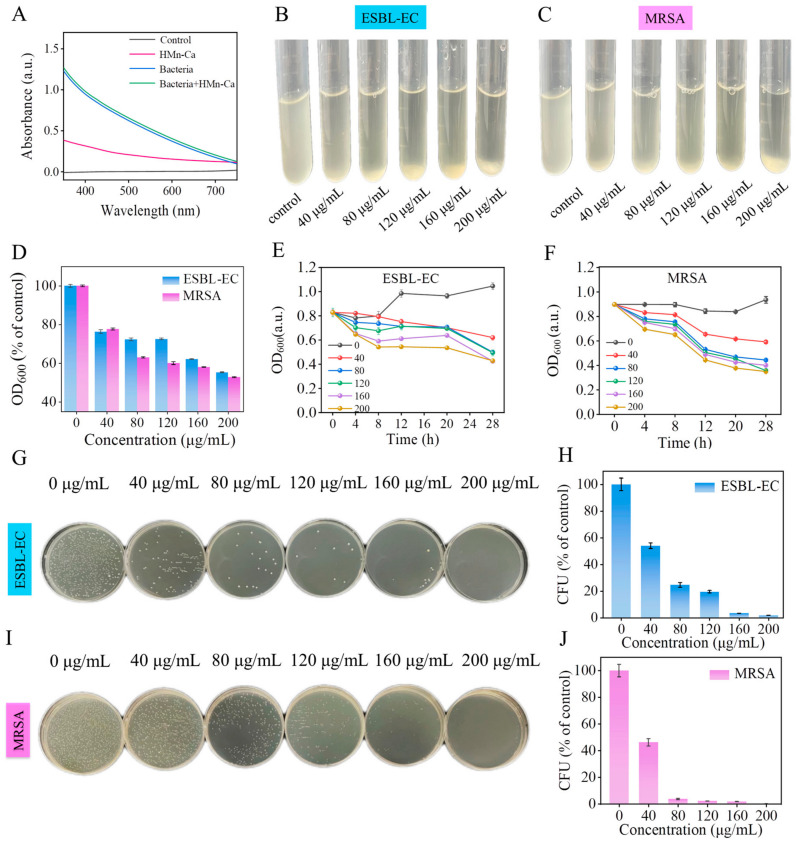
Antibacterial effects of HMn-Ca in vitro. (**A**) UV-vis absorbance of the material. (**B**,**C**) Photographs and (**D**) absorbance at 600 nm of ESBL-EC and MRSA after incubation with different concentrations of HMn-Ca. (**E**,**F**) The antibacterial activity of HMn-Ca shows absorbance changes at 600 nm, which are dependent on time. (**G**,**I**) Agar plate photographs and (**H**,**J**) the corresponding living bacteria numbers of ESBL-EC and MRSA after treatments with HMn-Ca at different concentrations.

Considering the bacterial absorption at 600 nm reflects the turbidity of the bacterial solution, we further assessed the antibacterial potential of HMn-Ca by conducting a growth-inhibition assay in bacterial solutions and measuring the UV-visible absorption of bacterial suspensions after treatment with HMn-Ca. First, we examined the UV-visible absorption of HMn-Ca and bacterial suspension. As shown in Figure 3A, the HMn-Ca solution showed weak UV-visible absorption, while solutions of MRSA and ESBL-EC displayed strong absorption at 600 nm. Moreover, mixing the solution of HMn-Ca with the solutions of MRSA and ESBL-EC had no effects on the UV-visible absorption of these bacterial suspensions, indicating that HMn-Ca could not directly affect the UV-visible absorption of bacteria. This allows us to discern the antimicrobial performance of HMn-Ca by evaluating the absorption values of the solution following the incubation of the material with the bacterial suspension. Next, we found that treatment with HMn-Ca nanoparticles reduced the turbidity and the absorbance (OD at 600 nm) of the ESBL-EC and MRSA culture medium in a dose- and time-dependent manner (Figure 3B–F), demonstrating the antibacterial ability of HMn-Ca. Additionally, we also examined the antibacterial ability of HMn-Ca against ESBL-EC and MRSA by the classical plate count method. As shown in Figure 3G–J, HMn-Ca nanoparticles showed extremely efficient bacterial killing activity on both ESBL-EC and MRSA. Taken together, these results demonstrated the anti-bacterial activity of HMn-Ca on antibiotic-resistant MRSA and ESBL-EC.

Notably, HMn-Ca exhibited greater efficacy in eliminating MRSA than ESBL-EC (Figure 3H,J). Almost all the MRSA were eradicated when treated with 200 μg/mL HMn-Ca, whereas a few ESBL-EC remained alive, further evidencing the better selectivity of HMn-Ca against MRSA. One potential explanation for this phenomenon is that MRSA may exhibit a higher negative surface potential, resulting in a stronger electrostatic attraction with HMn-Ca. Another contributing factor could be attributed to the spherical morphology of MRSA in contrast to the rod-shaped structure of ESBL-EC. The greater curvature associated with the spherical structure implies a larger contact area with the material, thereby leading to a higher adhesive propensity; thus, the adsorption effects of HMn-Ca on MRSA are more potent.

**Figure 4:**
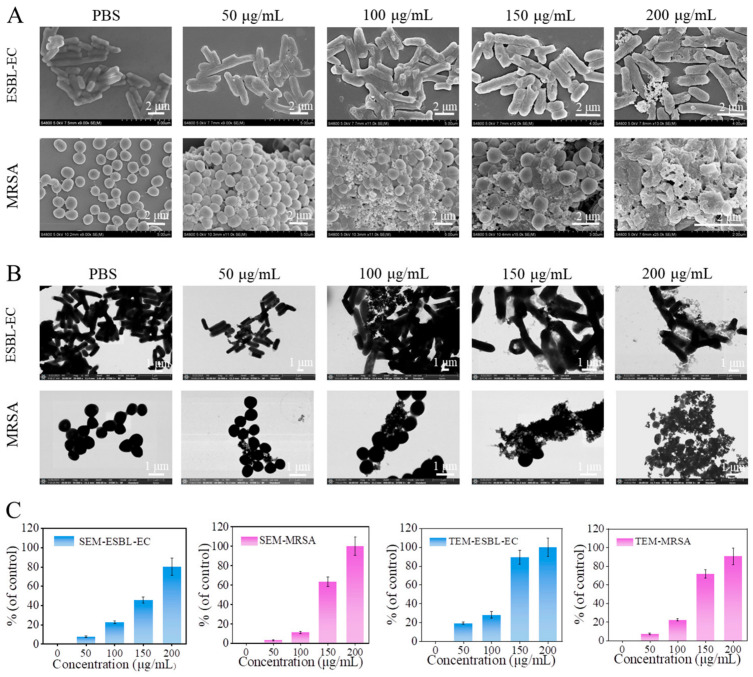
Bacterial capturing and bacterial membrane damaging capacity of HMn-Ca. (**A**) SEM and (**B**) TEM images of ESBL-EC and MRSA after incubation with different concentrations of HMn-Ca. (**C**) The bacteria capture rate of HMn-Ca quantified by SEM and TEM.

To further study the antibacterial mechanism of HMn-Ca, the interaction between bacteria and HMn-Ca was investigated by SEM and TEM. As illustrated in Figure 4A, it is observable that the bacteria in the control group exhibit a smooth, rod-like morphology. However, in the experimental group, with increasing concentrations of HMn-Ca, ESBL-EC rods experience fragmentation, accompanied by the development of surface wrinkles. MRSA also undergoes significant morphological deformations, displaying surface wrinkles and eventual rupture. At a concentration of 200 μg/mL, nearly all MRSA exhibit rupture. The data obtained from both SEM and TEM analyses are in complete agreement, providing evidence of the significant antimicrobial efficacy of HMn-Ca (Figure 4A,B). These results suggested that HMn-Ca had a strong adsorption and killing effect on bacteria, which may be due to the rough surface and ROS-generating efficiency of HMn-Ca. Additionally, when considering the conclusions drawn from UV-visible spectrophotometry and plate counting methods, a comparison reveals a superior antimicrobial effect against MRSA to ESBL-EC. Consequently, we proceeded to establish a wound infection model using MRSA for subsequent investigations.

### 2.3. HMn-Ca Accelerated Wound Healing in MRSA-Infected Mice 

**Figure 5:**
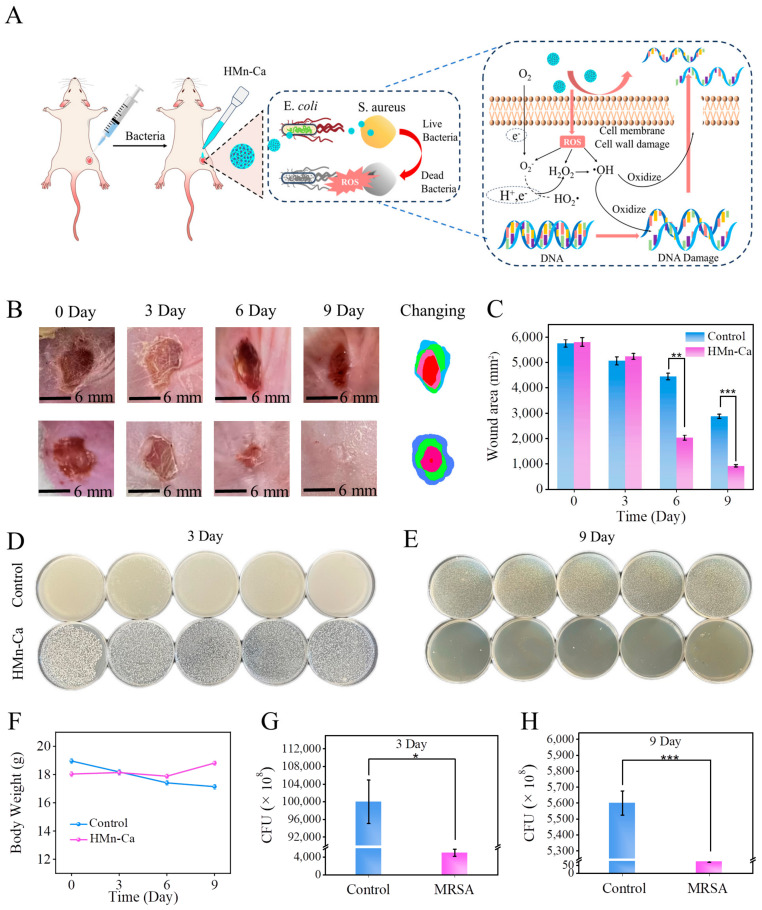
Antibacterial effects of HMn-Ca in vivo. (**A**) Experimental design for evaluating the antibacterial effects of HMn-Ca in mice. (**B**) Macroscopic views of the infected wound and (**C**) the corresponding wound area on days 0, 3, 6, and 9. Images of bacterial colonies rolled by MRSA of the wound tissue on (**D**) day 3 and (**E**) day 9. * *p* < 0.05, ** *p* < 0.01, *** *p* < 0.001. (**F**) The body weight of mice administered with PBS versus HMn-Ca. (**G**,**H**) Quantitative analysis of the relative bacteria viability rate in (**D**,**E**).

Encouraged by the above promising in vitro data, we further examined the effects of HMn-Ca on wound healing in MRSA-infected mice. The ICR mice with a back wound diameter of 6 mm were infected by MRSA, followed by treatment with PBS or HMn-Ca for 9 days. As shown in Figure 5A, gross observation of wound closure in mice suggested that HMn-Ca dramatically accelerated wound healing under MRSA infection when compared to the control group. The wound almost completely closed on day 9 in the HMn-Ca group, while obvious wound border and dermal incompleteness can still be seen in the control group (Figure 5B,C). Furthermore, bacterial infection significantly reduced the weight of mice compared to the control group, whereas the reduction was markedly reversed by HMn-Ca (Figure 5F). To test the anti-bactericidal property of HMn-Ca, the exudate samples were collected from the wound tissues on days 3 and 9, followed by incubation in the trypticase soy agar culture medium for 24 h. As shown in Figure 5D,E, the sample from the control group contained high levels of bacteria; administration with HMn-Ca effectively reduced the number of bacteria in the skin tissues.

### 2.4. Biocompatibility of HMn-Ca

**Figure 6:**
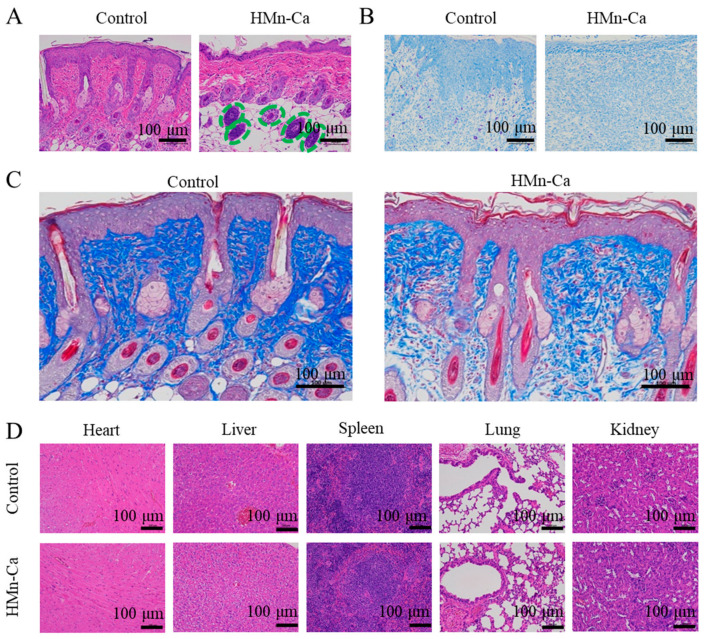
HMn-Ca promoted wound healing in mice. The skin lesions from the upper back of mice were subjected to (**A**) H&E staining (purple part is hair follicle tissue). (**B**) Giemsa staining (MRSA is dyed purple and normal tissue is blue), and (**C**) Masson’s trichrome staining (blue part is fibrin). The newborn hair follicles from the H&E staining image were indicated by green circles. (**D**) After 9 days of treatment, images of H&E staining were obtained for the major organs (heart, liver, spleen, lung, and kidney).

Next, hematoxylin and eosin (H&E) staining was employed to examine the effects of HMn-Ca on the healing pathology of wounds. Compared to the control group, thickened new epidermis, abundant granulation tissue, and hair follicles (indicated by green circles) were found in the wound gap of the HMn-Ca group on day 9. In contrast, more inflammatory cells, marked hemorrhage, and edema still emerged in the PBS control group (Figure 6A). Further, Giemsa staining was conducted to analyze the residual bacteria 9 days after therapy in vivo; more MRSA were found in the PBS control group than in the HMn-Ca-treated group, indicating the efficient bacterial killing capability of HMn-Ca in vivo (Figure 6B). Masson’s trichrome staining was also conducted to examine the collagen deposition in the wound-healing process. On Day 9, the wound of the HMn-Ca group expressed favorable established collagen fibers and dermal layer, while the PBS group displayed fewer collagen depositions (Figure 6C). Additionally, no appreciable abnormalities or injuries to major organs in the mice were found 9 days after treatment with HMn-Ca (Figure 6D), indicating the tissue biocompatibility of HMn-Ca. These findings suggested that HMn-Ca exhibited excellent anti-bactericidal efficiency against MRSA and could accelerate infection-related wound healing in vivo.

The therapeutic efficacy of HMn-Ca on MRSA infection-induced non-healing wounds may be dependent in two ways. First, MnO-mediated POD-like activity produces enough ROS, thus killing MRSA in the wound [57]. Second, calcium can modulate blood clotting by facilitating the formation of the platelet plug, increasing the proliferation, migration, and collagen synthesis of fibroblasts, as well as promoting angiogenesis [58]. Furthermore, it has been reported that wound healing is slow in animals with dietary calcium deficiency or with calcium chelating agents in their diet [59]. These results, combined with the present study, suggest the important role of calcium in wound healing.

Moreover, Victoria M. et al. have reported calcium phosphate nanoparticles as antimicrobials, which exhibited potent inhibitory activity against Gram-positive bacteria but had poor effects on Gram-negative bacteria [60]. Talebpour et al. have developed AgNbO_3_ nanoparticles that could prevent the proliferation of both Gram-positive *Staphylococcus aureus* and Gram-negative *Pseudomonas aeruginosa*. However, it is nondegradable, possessing a certain degree of destructiveness and environmental contamination [61]. The HMn-Ca prepared in our study effectively leverages the advantages of calcium and manganese, demonstrating the ability to inhibit both Gram-negative and Gram-positive bacteria while also promoting wound healing in bacterial-infected wounds.

## 3. Conclusions 

To develop antibacterial materials for antibiotic-resistant bacterial infections and chronic non-healing wounds, we have successfully synthesized mesoporous silicon loaded with MnO and CaO molecules (HMn-Ca). The preparation method is straightforward, utilizing precursor materials readily available from diverse sources, resulting in low production costs and high material yields. The materials were characterized for size and structure using SEM and TEM, revealing that HMn-Ca possesses uniform pores and diameters. Due to its high specific surface area, multiple pore sizes, and elevated adhesion capability, generating ROS and facilitating the promotion of hair follicles, the developed HMn-Ca exhibits great potential for bacteria capture and killing. UV-visible spectrophotometry experiments demonstrated that HMn-Ca exhibits inhibition of ESBL-EC and MRSA bacteria, respectively. In a mouse bacterial wound model, wounds treated with HMn-Ca achieved a wound closure rate of 98.71% on the 9th day, likely due to its strong adhesive properties and its capacity to promote ROS production to kill bacteria and facilitate normal tissue follicle growth.

As a result, treatment with HMn-Ca could improve the healing of the bacterial-infected wound, demonstrating a straightforward yet potent method for engineering nanozymes for antibacterial therapy. Furthermore, our results highlighted the promising potential of HMn-Ca in treating infection and diabetes-induced chronic non-healing wounds, as well as improving functional outcomes and quality of life.

## 4. Experimental

### 4.1. Materials Synthesis 

We procured all the reagents utilized in this study from Sinopharm (Shanghai, China). Luria-Bertani (LB) liquid medium was purchased from Solarbio. Transmission electron microscope (TEM) images and energy dispersive X-ray (EDX) spectroscopy were performed by field emission high-resolution transmission electron microscopy (JEOL, Peabody, MA, USA). Scanning electron microscopy (SEM) images were obtained with a field emission scanning electron microscope (Sigma, Jena, Germany). The Mercury 3.0 program was used to simulate diffraction patterns. Optical diffuse reflectance and UV spectra were recorded at room temperature on an Agilent Cary 5000 UV-Vis-NIR spectrophotometer (Agilent, Santa Clara, CA, USA). Nitrogen absorption/desorption analysis at 77K were carried out using a H-Sorb 2600 volumetric absorption analyzer (H-Sorb, Hefei, China). The ZetaSizer Nano ZS90 (Malvern, Makvern, UK) was used to measure the z-average hydrodynamic diameter of nanoparticles based on dynamic light scattering (DLS) and zeta potential based on electrophoretic mobility (ICPMS-2030 (Shimadzu, Kyoto, Japan)).

### 4.2. Synthesis and Characterization of HMn-Ca

Mesoporous silica nanospheres (MSNs) were prepared through a one-pot synthesis process [62]. Tetraethyl orthosilicate (4.7 M) in cyclohexane (16 mL) was added dropwise to a mixture of hexadecyltrimethylammonium bromide (CTAB) (1.5 g) and Et_3_N (5 M) in deionized water (60 mL). The resulting solution was stirred at 60 °C for 48 h (pH = 7.0) and centrifuged at 8000 r/min for 5 min. The supernatant containing excess reagents and impurities was carefully removed. The pellet, which included MSNs and some residual substances, was washed with ethanol (5 × 10 mL) to eliminate any residual unreacted remnants and resuspended with ethanol (20 mL). The obtained MSN solutions were used as such for the next reaction. Subsequently, 2 mL of the obtained MSN solutions were added dropwise to a mixture of MnCl_2_ (0.08 g) and CaCl_2_ (0.02 g) in deionized water (60 mL). After stirring at 90 °C for 35 min, a solution of urotropine (0.1 g) in deionized water (5 mL) was slowly added. The reaction blend was stirred at 90 °C for 2 h. After centrifugation at 2000× *g* for 30 min, the obtained HMn-Ca loading with MnO and CaO molecules at a percentage of 16.44 wt% and 2.11 wt%, respectively, and the remainder comprising deionized water, was followed by drying at 60 °C for 12 h. The morphology and particle size of MSNs and HMn-Ca were characterized by SEM and TEM. The prepared sample was dispersed in pure ethanol and ultrasonicated for 15 min. After that, a diluted sample was added dropwise onto the silicon wafer, dried for SEM testing, and poured onto a holey carbon-coated copper grid and left to dry before TEM analysis.

### 4.3. Physical Characterization

The chemical surface species of HMn-Ca was tested using X-ray photoelectron spectrometry (XPS). The stability of HMn-Ca was investigated by measuring the dissolution of Mn and Ca in deionized water. Briefly, HMn-Ca (0.5 mg) was stirred in deionized water (100 mL) for 72 h, followed by centrifugation at 8000 r/min for 5 min. The concentrations of Mn and Ca were measured by the on-line inductive coupled plasma mass spectrometer (ICP-MS) (ULTIMA 2, Horiba Jobin Yvon S.A.S., Paris, France).

Nitrogen absorption/desorption analysis was conducted utilizing a nitrogen absorption apparatus at 77 K. Subsequently, the samples underwent a mass of 200 mg and degassing at 300 °C under a nitrogen atmosphere. The specific surface area was determined using the BET method. The BET method was calculated by employing the same procedures typically used for experimental measurements when analyzing adsorption isotherms.

The linearized BET isotherm, derived from the BET theory, was utilized in performing the BET analysis.

(1) $$\frac{P/P_{0}}{N\left(1-P/P_{0}\right)}=\frac{1}{N_{m}C}+\frac{C-1}{N_{m}C}\left(\frac{P}{P_{0}}\right)$$

Here, *P*_0_ refers to the saturation pressure (1 bar), and *P*/*P*_0_ denotes the relative pressure.

Constants *C* and *Nm* pertain to the energetics of adsorption and the loading of the monolayer, respectively. *N* signifies the amount of adsorbate loaded, which can be obtained.

### 4.4. ICPMS

To assess the stability of the material, it was subjected to analysis via inductively coupled plasma mass spectrometry. Accurately weigh 0.2 mg of dried HMn-Ca (precisely to 0.0001 g), followed by the addition of 10 mL of PBS (phosphate buffered saline, a common biological buffering solution frequently employed for the dissolution or dilution of biological agents with a pH of 6.4). After incubating in the solution for 72 h, followed by centrifugation, the concentrations of dissolved manganese and calcium ions were determined via ICP analysis.

### 4.5. Particle Size and Zeta Potential Assay

Diameter and zeta potential were measured by the NanoBrook Omni particle sizer and zeta potential analyzer according to the manufacturer’s instructions [62]. After dilution with 5000 times deionized water (*v*/*v*), 1 mL of the diluted bacterial suspension and HMn-Ca solution were used for the Zeta potential assay. To examine the interaction between HMn-Ca and bacteria, HMn-Ca (200 µg/mL) was incubated in the bacterial suspensions (1 × 10^8^ CFU/mL, 200 μL) at 37 °C for 2 h, followed by centrifugation at 6500 rpm for 5 min. After washing with deionized water 3 times, the cell pellets were suspended in 3 mL of deionized water and then analyzed by the Dynamic Light Scattering Instrument.

### 4.6. Morphology Observation of Bacteria

After incubation with PBS and HMn-Ca (200 µg/mL) at 37 °C for 30 min, the bacteria (1 × 10^8^ CFU/mL) were fixed in a solution of 10% paraformaldehyde at 4 °C overnight. The samples were transferred through a range of ethanol solutions, with concentrations ranging from 30% to 100%, to gradually dehydrate the specimens. The dehydrated bacterial samples were centrifuged at 6500 rpm for 8 min, and the dried bacteria were collected and used as such for the SEM and TEM studies.

### 4.7. Generation of Reactive Oxygen Species (ROS)

The POD-like activity of HMn-Ca was determined according to a previously reported method [57]. A 200 μL H_2_O_2_ solution (final concentration 0.5, 1.0, and 2.0 mM) was added to a solution of HMn-Ca (100 μg/mL) and 3,3′,5,5′-tetramethylbenzidine (TMB) (2.4 mM) in a phosphate buffer solution (PBS, 100 μL, pH 6.4). At 37 °C for 30 min, incubation of the resulting mixture occurred. ROS generation was investigated through measurement of the absorption changes of the oxidized form of TMB at 652 nm.

### 4.8. Growth-Inhibition Assay in Liquid Medium

Preparation of Luria-Bertani (LB) Liquid Medium: Tryptone (2.5 g), sodium chloride (2.5 g), and yeast extract (1.25 g) were simultaneously dissolved in 250 mL of deionized water (DI). Subsequently, the mixture’s pH was set to 7.5 with a solution of NaOH. The solution was then sterilized at 120 °C for 30 min. Finally, a light yellow, clear solution was collected, and the prepared LB medium was stored at 4 °C.

Gram-positive methicillin-resistant *Staphylococcus aureus* (MRSA, ATCC, Cat. # 700698) and Gram-negative extended-spectrum beta-lactamase (ESBL)-producing *Escherichia coli* (ESBL-EC, ATCC, Cat. # BAA-196) were selected as the experimental strains to evaluate the antibacterial characteristics of the HMn-Ca. In the bacterial suspensions of MRSA (1 × 10^8^ CFU/mL, 200 μL) or ESBL-EC (1 × 10^8^ CFU/mL, 200 μL) in LB liquid medium (5 mL), various concentrations of HMn-Ca (0, 40, 80, 120, 160, and 200 µg/mL) were introduced. Subsequently, the mixture underwent a 4-h incubation at 37 °C before measuring the absorbance at 600 nm. Each treatment was analyzed with at least 3 replicates.

### 4.9. Plate Counting Method

Preparation of Solid LB Medium: Tryptone (5 g), sodium chloride (5 g), and yeast extract (2.5 g) were dissolved simultaneously in 500 mL of deionized water (DI). Then, 3.75 g of agar powder was incorporated into the solution. Following autoclaving and removal at 80 °C, the medium was poured into Petri plates on a sterile operation table. Each Petri dish was approximately 5 cm thick, and after cooling and solidification at room temperature, they were sealed with film and stored at 5 °C in a refrigerator.

The amount of bacterial colonies (CFU/mL) present in the suspension was measured using the plate counting method. Following a 10,000-fold dilution with regular LB liquid medium, 100 μL of the diluted bacterial suspension was plated on the solid medium and subjected to a 24-h incubation at 37 °C. Then the count of growing colonies (CFU) on the agar plates was determined. Each treatment underwent analysis with a minimum of 3 replicates.

### 4.10. In Vivo Wound Healing Model

All animal experiments adhered to the animal care guidelines. The protocols concerning animal study and welfare underwent review and approval by the Committee on the Ethics of Animal Experiments of the Chinese Academy of Science (approval number 2020-01-01JiPi). Cages with free access to food and water were provided for ICR mice (18–22 g), which were housed under a 12-h dark/light cycle and maintained at a temperature of 22 ± 3 °C. Isoflurane was used to anesthetize the mice, and their back cutaneous hair was removed via electrical shaving. Next, wounds with a diameter of 6 mm and full thickness were created utilizing a milter biopsy perforator, followed by injection of 100 μL of 1 × 10^8^ CFU mL^−1^ MRSA bacteriophage solution into the exposed wounds. One day after MRSA injection, HMn-Ca (2 μg in 100 μL PBS) was applied topically to skin wounds. Using a digital camera, photographs of the wounds were taken on days 0, 1, 3, 5, 7, and 9. The measurement of the wound area was conducted using Image J. On days 3 and 9, the bacterial sample from the wound area was collected by using sterile cotton swabs, and the solid medium was evenly spread, followed by an incubation period of 24 h at 37 °C. Each treatment was analyzed using at least 5 replicates.

### 4.11. Histology

Nine days after MRSA injection, wound tissues—heart, liver, spleen, lungs, and kidneys—were accumulated, fixed in 10% paraformaldehyde at 4 °C overnight, then underwent dehydration before being embedded in paraffin. Sections, each 5 µm thick, were cut using a microtome, then subjected to staining with hematoxylin and eosin, Masson trichrome, and Giemsa, followed by scanning using an optical microscope.

### 4.12. Data Validity

The data are presented as means ± standard error of the mean (SEM). Analyses were conducted using GraphPad Prism 9.0.5. Two groups were used and analyzed using the unpaired Student’s *t*-test with Welch’s correction for unequal variances. Significance was determined at *p* < 0.05. No data points or mice were excluded.

## Data Availability

The data presented in this study are available on request from the corresponding author due to legal restriction.

## Abbreviations

Methicillin-resistant *Staphylococcus aureus* (MRSA), extended-Spectrum β-Lactamases-producing *Escherichia coli* (ESBL-EC), phosphate buffer saline (PBS), 3,3′,5,5′-Tetramethylbenzidine (TMB), Triethylamine (Et3N), multi-drug resistance (MDR), reactive oxygen species (ROS), Mesoporous Silica Nanoparticles (MSNs), Institute of Cancer Research (ICR), hematoxylin–eosin (H&E).

## References

[1] Scott G.I., Porter D.E., Norman R.S., Scott C.H., Uyaguari-Diaz M.I., Maruya K.A., Weisberg S.B., Fulton M.H., Wirth E.F., Moore J. (2016). Antibiotics as CECs: An Overview of the Hazards Posed by Antibiotics and Antibiotic Resistance. Front. Mar. Sci..

[2] Beardmore R.E., Peña-Miller R., Gori F., Iredell J. (2017). Antibiotic Cycling and Antibiotic Mixing: Which One Best Mitigates Antibiotic Resistance?. Mol. Biol. Evol..

[3] Zhang R., Yang S., An Y., Wang Y., Lei Y., Song L. (2022). Antibiotics and antibiotic resistance genes in landfills: A review. Sci. Total Environ..

[4] Munoz-Price L.S., Frencken J.F., Tarima S., Bonten M. (2016). Handling Time-dependent Variables: Antibiotics and Antibiotic Resistance. Clin. Infect. Dis..

[5] Torraca V., Mostowy S. (2016). Septins and Bacterial Infection. Front. Cell Dev. Biol..

[6] Van Elsland D., Neefjes J. (2018). Bacterial infections and cancer. EMBO Rep..

[7] Upadhayay A., Ling J., Pal D., Kumar A. (2023). Resistance-proof antimicrobial drug discovery to combat global antimicrobial resistance threat. Drug Resist. Updates.

[8] Gao W., Chen Y., Zhang Y., Zhang Q., Zhang L. (2018). Nanoparticle-based local antimicrobial drug delivery. Adv. Drug Deliv. Rev..

[9] Chifiriuc M.C., Filip R., Constantin M., Pircalabioru G.G., Bleotu C., Burlibasa L., Ionica E., Corcionivoschi N., Mihaescu G. (2022). Common themes in antimicrobial and anticancer drug resistance. Front. Microbiol..

[10] Ye L., Zhang J., Xiao W., Liu S. (2020). Efficacy and mechanism of actions of natural antimicrobial drugs. J. Vet. Pharmacol. Ther..

[11] Dhivya S., Padma V.V., Santhini E. (2015). Wound dressings—A review. BioMedicine.

[12] Sader H.S., Rhomberg P.R., Fuhrmeister A.S., Mendes R.E., Flamm R.K., Jones R.N. (2019). Antimicrobial Resistance Surveillance and New Drug Development. Open Forum Infect. Dis..

[13] Yimeng S., Huilun X., Ziming L., Kejun L., Chaima M., Yinchun H., Yan W., Di H. (2023). Copper-Based Nanoparticles as Antibacterial Agents. Eur. J. Inorg. Chem..

[14] Shrestha A., Kishen A. (2016). Antibacterial Nanoparticles in Endodontics: A Review. J. Endod..

[15] Bhattacharya P., Neogi S. (2019). Antibacterial properties of doped nanoparticles. Rev. Chem. Eng..

[16] Huang X., Lu B., Zhao Y., Wang Z., Wang H., Yuan L. (2021). The Antibacterial Effect of Bacteriophage-Like Gold Nanoparticles. Nano Brief. Rep. Rev..

[17] Bruna T., Maldonado-Bravo F., Jara P., Caro N. (2021). Silver Nanoparticles and Their Antibacterial Applications. Int. J. Mol. Sci..

[18] Smirnov N.A., Kudryashov S.I., Nastulyavichus A.A., Rudenko A.A., Saraeva I.N., Tolordava E.R., Gonchukov S.A., Romanova Y.M., Ionin A.A., Zayarny D.A. (2018). Antibacterial properties of silicon nanoparticles. Laser Phys. Lett..

[19] Ciabocco M., Cancemi P., Saladino M.L., Caponetti E., Alduina R., Berrettoni M. (2018). Synthesis and antibacterial activity of iron-hexacyanocobaltate nanoparticles. J. Biol. Inorg. Chem..

[20] Qamer S., Romli M.H., Che-Hamzah F., Misni N., Joseph N.M., Al-Haj N.A., Amin-Nordin S. (2021). Systematic Review on Biosynthesis of Silver Nanoparticles and Antibacterial Activities: Application and Theoretical Perspectives. Molecules.

[21] Lv P., Zhu L., Yu Y., Wang W., Liu G., Lu H. (2020). Effect of NaOH concentration on antibacterial activities of Cu nanoparticles and the antibacterial mechanism. Mater. Sci. Eng. C-Mater..

[22] Precious Ayanwale A., Reyes-López S.Y. (2019). ZrO_2_-ZnO Nanoparticles as Antibacterial Agents. ACS Omega.

[23] Harvey N.C., Biver E., Kaufman J.M., Bauer J., Branco J., Brandi M.L., Cooper C. (2017). The role of calcium supplementation in healthy musculoskeletal ageing: An expert consensus meeting of the European Society for Clinical and Economic Aspects of Osteoporosis, Osteoarthritis and Musculoskeletal Diseases (ESCEO) and the International Foundat. Osteoporosis Int..

[24] Tang N.H., Kim K.W., Xu S., Blazie S.M., Yee B.A., Yeo G.W., Jin Y., Chisholm A.D. (2020). The mRNA decay factor CAR-1/LSM14 regulates axon regeneration via mitochondrial calcium dynamics. Curr. Biol..

[25] Pirouz M., Wang C.H., Liu Q., Ebrahimi A.G., Shamsi F., Tseng Y.H., Gregory R.I. (2020). The Perlman syndrome DIS3L2 exoribonuclease safeguards endoplasmic reticulum-targeted mRNA translation and calcium ion homeostasis. Nat. Commun..

[26] Molina-Hernandez J.B., Aceto A., Bucciarelli T., Paludi D., Valbonetti L., Zilli K., Scotti L., Chaves-López C. (2021). The membrane depolarization and increase intracellular calcium level produced by silver nanoclusters are responsible for bacterial death. Sci. Rep..

[27] Liu S., Zheng Z., Wang S., Chen S., Ma J., Liu G., Li J. (2019). Polydopamine-coated chitosan/calcium pyrophosphate hybrid microflowers as an effective hemostatic agent. Carbohydr. Polym..

[28] Abinaya S., Kavitha H.P. (2023). Magnesium oxide nanoparticles: Effective antilarvicidal and antibacterial agents. ACS Omega.

[29] Davidson E., Pereira J., Gan Giannelli G., Murphy Z., Anagnostopoulos V., Santra S. (2023). Multi-Functional chitosan nanovesicles loaded with bioactive manganese for potential wound healing applications. Molecules.

[30] Liu L., Wang C., Li Y., Qiu L., Zhou S., Cui P., Jiang P., Ni X., Liu R., Du X. (2021). Manganese dioxide nanozyme for reactive oxygen therapy of bacterial infection and wound healing. Biomater. Sci..

[31] Mesaros A., Vasile B.S., Toloman D., Pop O.L., Marinca T., Unguresan M., Perhaita I., Filip M., Iordache F. (2019). Towards understanding the enhancement of antibacterial activity in manganese doped ZnO nanoparticles. Appl. Surf. Sci..

[32] Radhi Devi K., Bruno Chandrasekar L., Kasirajan K., Karunakaran M., Divya Gnaneswari M., Usha S. (2022). Enhanced in vitro antibacterial activity of ZnO and Mn–Mg co-doped ZnO nanoparticles: Investigation of synthesis, characterization, and impact of dopant. Appl. Phys. A.

[33] Jimenez J., Chakraborty I., Carrington S.J., Mascharak P.K. (2016). Light-triggered CO delivery by a water-soluble and biocompatible manganese photoCORM. Dalton Trans..

[34] Gao F., Sun M., Zhang J., Chang Y., Gao W., Ma G., Guo Y. (2022). Fenton-like reaction and glutathione depletion by chiral manganese dioxide nanoparticles for enhanced chemodynamic therapy and chemotherapy. J. Colloid Interface Sci..

[35] Li H., Gao Q., Wang G., Han B., Zhou C. (2020). Unique electron reservoir properties of manganese in Mn(II)-doped CeO_2_ for reversible electron transfer and enhanced Fenton-like catalytic performance. Appl. Surf. Sci..

[36] Zhu C., Ma Q., Gong L., Di S., Gong J., Wang Y., Lin Z. (2022). Manganese-based multifunctional nanoplatform for dual-modal imaging and synergistic therapy of breast cancer. Acta Biomater..

[37] Chen Y., Chen M., Zhai T., Zhou H., Zhou Z., Liu X., Yang S., Yang H. (2022). Glutathione-Responsive Chemodynamic Therapy of Manganese(III/IV) Cluster Nanoparticles Enhanced by Electrochemical Stimulation via Oxidative Stress Pathway. Bioconjug. Chem..

[38] Kurtan U., Güner A., Amir M.D., Baykal A. (2017). Enhanced antibacterial performance of Fe_3_O_4_–Ag and MnFe_2_O_4_–Ag nanocomposites. Bull. Mater. Sci..

[39] Hartati H., Subaer S., Hasri H., Wibawa T., Hasriana H. (2022). Microstructure and Antibacterial Properties of Chitosan-Fe_3_O_4_-AgNP Nanocomposite. J. Nanomater..

[40] Wang D., Han S., Dai X. (2022). AIEgens functionalized hollow mesoporous silica nanospheres for selective detection of the antimicrobial furazolidone. Inorg. Chem. Commun..

[41] Chen J., Wei Y., Yang X., Ni S., Hong F., Ni S. (2020). Construction of selenium-embedded mesoporous silica with improved antibacterial activity. Colloids Surf. B.

[42] Wang Y., Wang Y., Su L., Luan Y., Du X., Zhang X. (2019). Effect of surface topology morphologies of silica nanocarriers on the loading of Ag nanoparticles and antibacterial performance. J. Alloys Compd..

[43] Li Y., Yan Y., Wang J., Li L., Tang F. (2022). Preparation of silver nanoparticles decorated mesoporous silica nanorods with photothermal antibacterial property. Colloids Surf. A.

[44] Yan Y., Liu Y., Li J., Li Y., Wu H., Li H., Ma X., Tang Y., Tong Y., Yi K. (2023). A Molecular Switch-Integrated Nanoplatform Enables Photo-Unlocked Antibacterial Drug Delivery for Synergistic Abscess Therapy. Adv. Healthc. Mater..

[45] Wang Y., Yin M., Lin X., Li L., Li Z., Ren X., Sun Y. (2019). Tailored synthesis of polymer-brush-grafted mesoporous silicas with N-halamine and quaternary ammonium groups for antimicrobial applications. J. Colloid Interface Sci..

[46] He X., Chen F., Chang Z., Waqar K., Hu H., Zheng X., Wang Y., Dong W.F., Yang C. (2022). Silver Mesoporous Silica Nanoparticles: Fabrication to Combination Therapies for Cancer and Infection. Chem. Rec..

[47] Michailidis M., Sorzabal-Bellido I., Adamidou E.A., Diaz-Fernandez Y.A., Aveyard J., Wengier R., Grigoriev D., Raval R., Benayahu Y., D’Sa R.A. (2017). Modified Mesoporous Silica Nanoparticles with a Dual Synergetic Antibacterial Effect. ACS Appl. Mater. Interfaces.

[48] Du J., Sun J., Liu X., Wu Q., Shen W., Gao Y., Liu Y., Wu C. (2023). Preparation of C6 cell membrane-coated doxorubicin conjugated manganese dioxide nanoparticles and its targeted therapy application in glioma. Eur. J. Pharm. Sci..

[49] Wu Z., Zhuang H., Ma B. (2021). Manganese-Doped Calcium Silicate Nanowire Composite Hydrogels for Melanoma Treatment and Wound Healing. Research.

[50] Haag S.L., Schiele N.R., Bernards M.T. (2022). Enhancement and mechanisms of MC3T3-E1 osteoblast-like cell adhesion to albumin through calcium exposure. Biotechnol. Appl. Biochem..

[51] Kim Y.R., Lee S.E., Kang I.C., Nam K.I., Choy H.E., Rhee J.H. (2013). A bacterial RTX toxin causes programmed necrotic cell death through calcium-mediated mitochondrial dysfunction. J. Infect. Dis..

[52] Festjens N., Vanden Berghe T., Vandenabeele P. (2006). Necrosis, a well-or-chestrated form of cell demise: Signalling cascades, important media- tors and concomitant immune response. Biochim. Biophys. Acta..

[53] Golstein P., Kroemer G. (2007). Cell death by necrosis: Towards a molecular definition. Trends. Biochem. Sci..

[54] Chang Z., Wang Z., Lu M., Li M., Li L., Zhang Y., Shao D., Dong W. (2017). Magnetic Janus nanorods for efficient capture, separation and elimination of bacteria. RSC Adv..

[55] Astuti S.D., Puspita P.S., Putra A.P., Zaidan A.H., Fahmi M.Z., Syahrom A., Suhariningsih (2019). The antifungal agent of silver nanoparticles activated by diode laser as light source to reduce *C. albicans* biofilms: An in vitro study. Lasers Med. Sci..

[56] Wilson W.W., Wade M.M., Holman S.C. (2001). Status of methods for assessing bacterial cell surface charge properties based on zeta potential measurements. J. Microbial. Meth..

[57] Chi Z.L., Yu G.H., Kappler A., Liu C.Q., Gadd G.M. (2021). Fungal–mineral interactions modulating intrinsic peroxidase-like activity of iron nanoparticles: Implications for the biogeochemical cycles of nutrient elements and attenuation of contaminants. Environ. Sci. Technol..

[58] Liu Y., Wang J. (2023). Multivalent metal catalysts in Fenton/Fenton-like oxidation system: A critical review. Chem. Eng. J..

[59] He J., Zheng Z., Lo I.M. (2021). Different responses of gram-negative and gram-positive bacteria to photocatalytic disinfection using solar-light-driven magnetic TiO_2_-based material, and disinfection of real sewage. Water Res..

[60] Wu V.M., Tang S., Uskokovic V. (2018). Calcium phosphate nanoparticles as intrinsic inorganic antimicrobials: The antibacterial effect. ACS Appl. Mater. Interfaces.

[61] Talebpour C., Fani F., Ouellette M., Salimnia H., Alamdari H. (2022). Nondegradable Antimicrobial Silver-Based Perovskite. ACS Sustain. Chem. Eng..

[62] Schwechheimer C., Kuehn M.J. (2015). Outer-membrane vesicles from Gram-negative bacteria: Biogenesis and functions. Nat. Rev. Microbial..

